# Supplementary material for: A Preliminary Study of Mild Heat Stress on Inflammasome Activation in Murine Macrophages
Source: Cells. 2023 Apr 19;12(8):1189. doi: 10.3390/cells12081189 (PMC10137183; doi:10.3390/cells12081189)
Supplement: Supplementary file 1 [file cells-12-01189-s001.zip › cells-2102583-supplementary.pdf]

Supplemental FIGURE S1 Related to Figure 2.

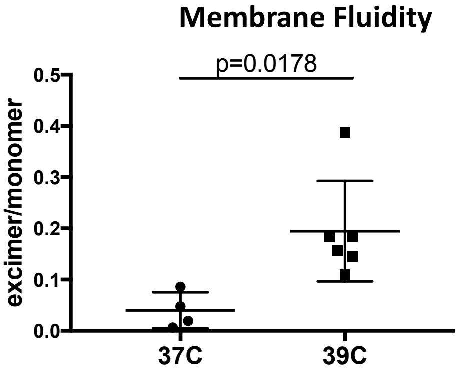

Figure S1. Exposure to 39°C increases membrane fluidity. Results are representative of 2 independent experiments.

Supplemental FIGURE S2—uncropped western blots related to FIGURE 3A

IL-1β

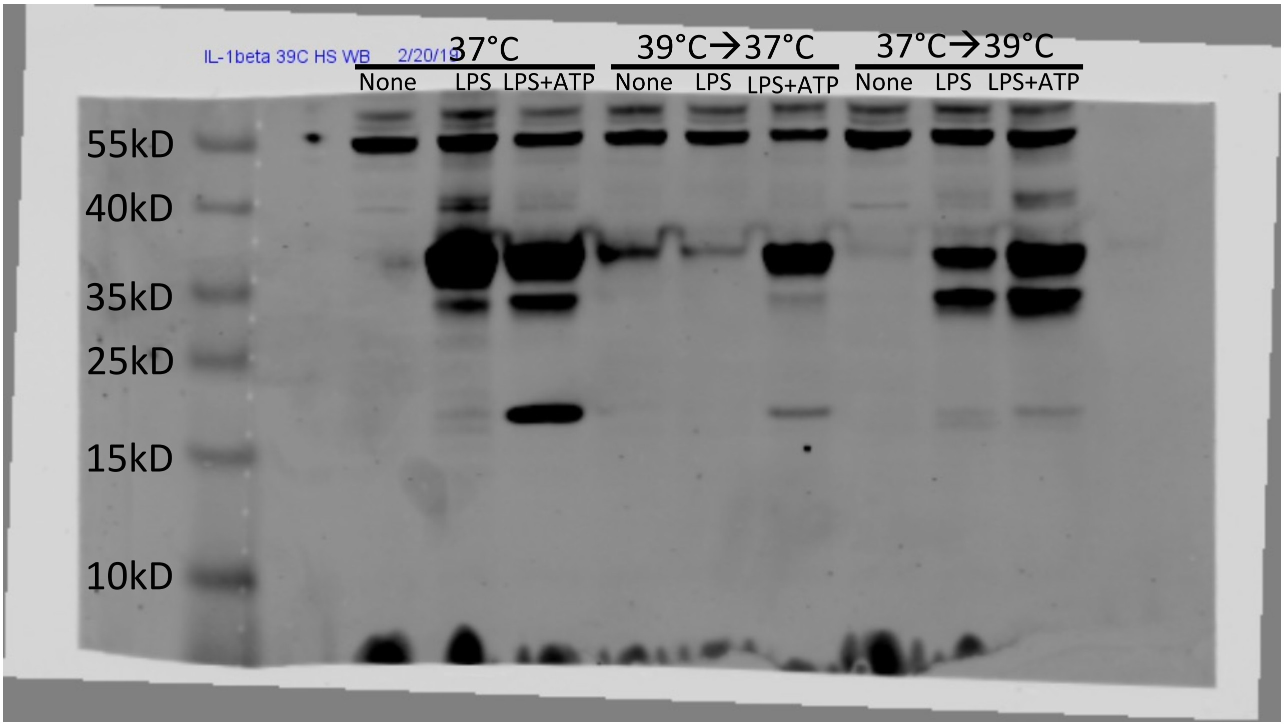

Caspase 1

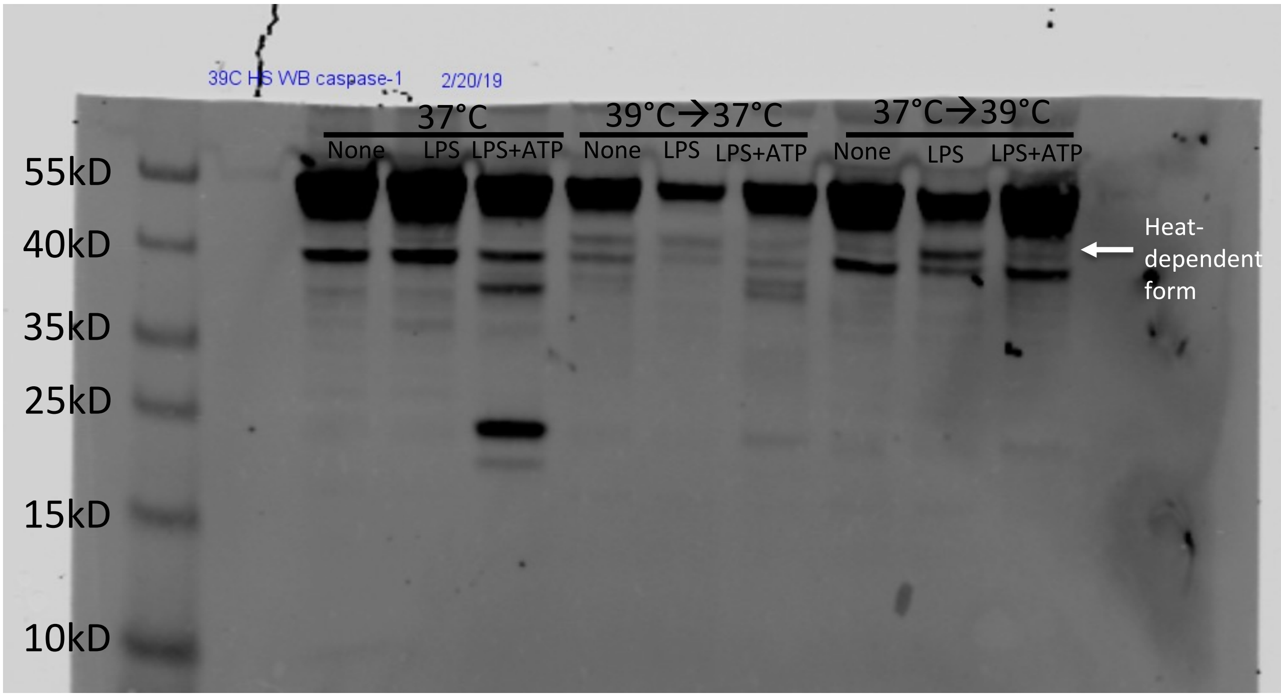

GAPDH

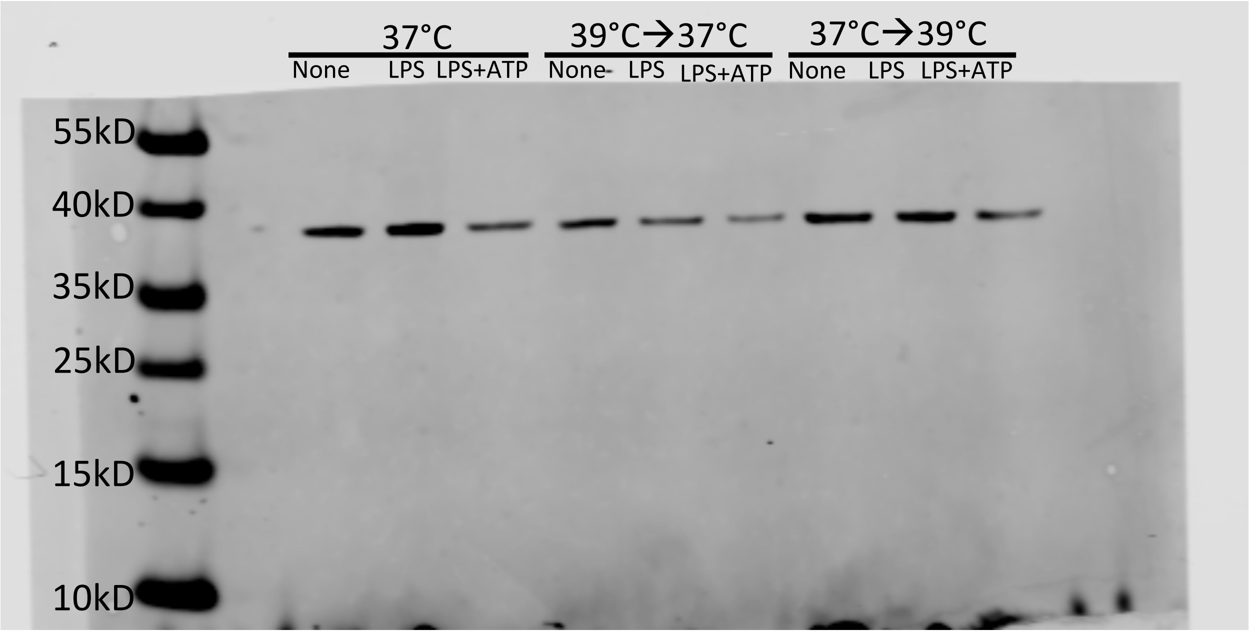
37°C
39°C→37°C
37°C→39°C
None
LPS
LPS+ATP
None
LPS
LPS+ATP
None
LPS
LPS+ATP
55kD
40kD
35kD
25kD
15kD
10kD

Supplemental FIGURE S3—uncropped western blots related to FIGURE 3B

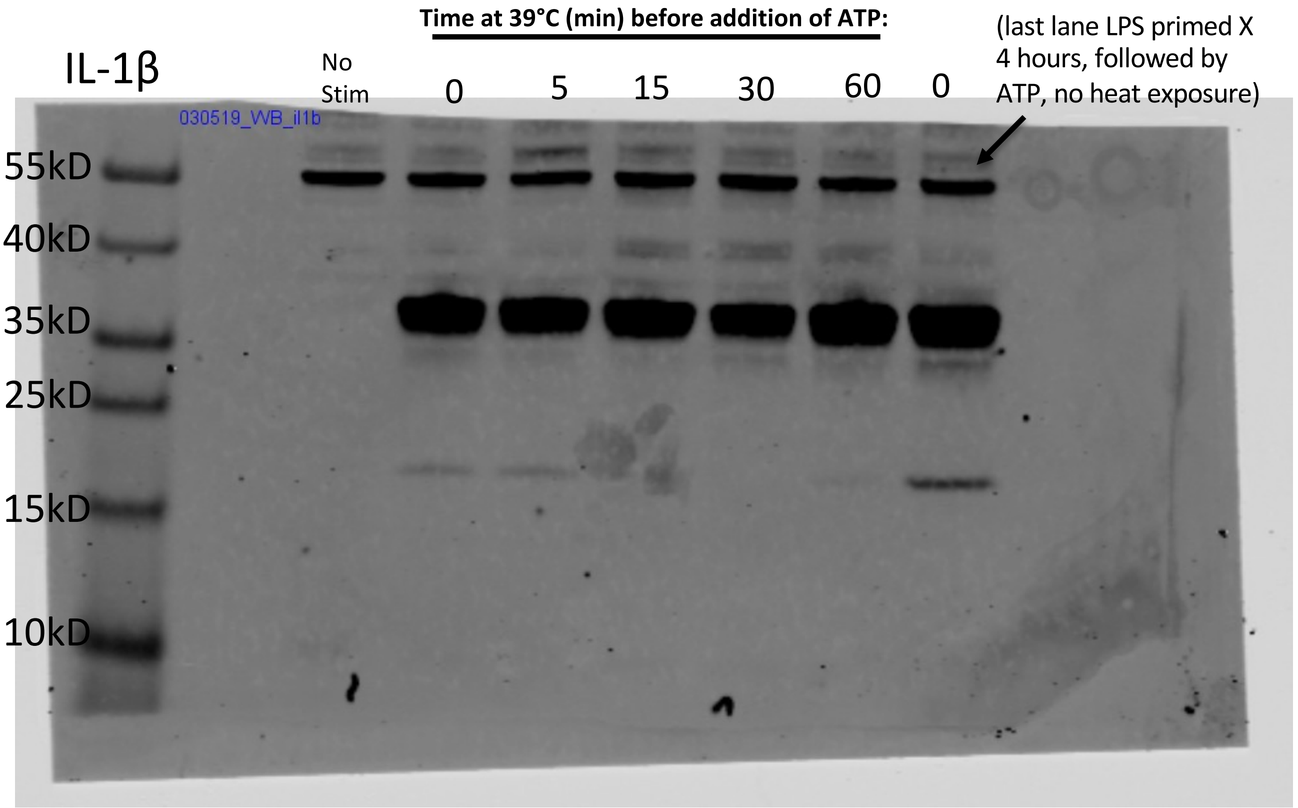

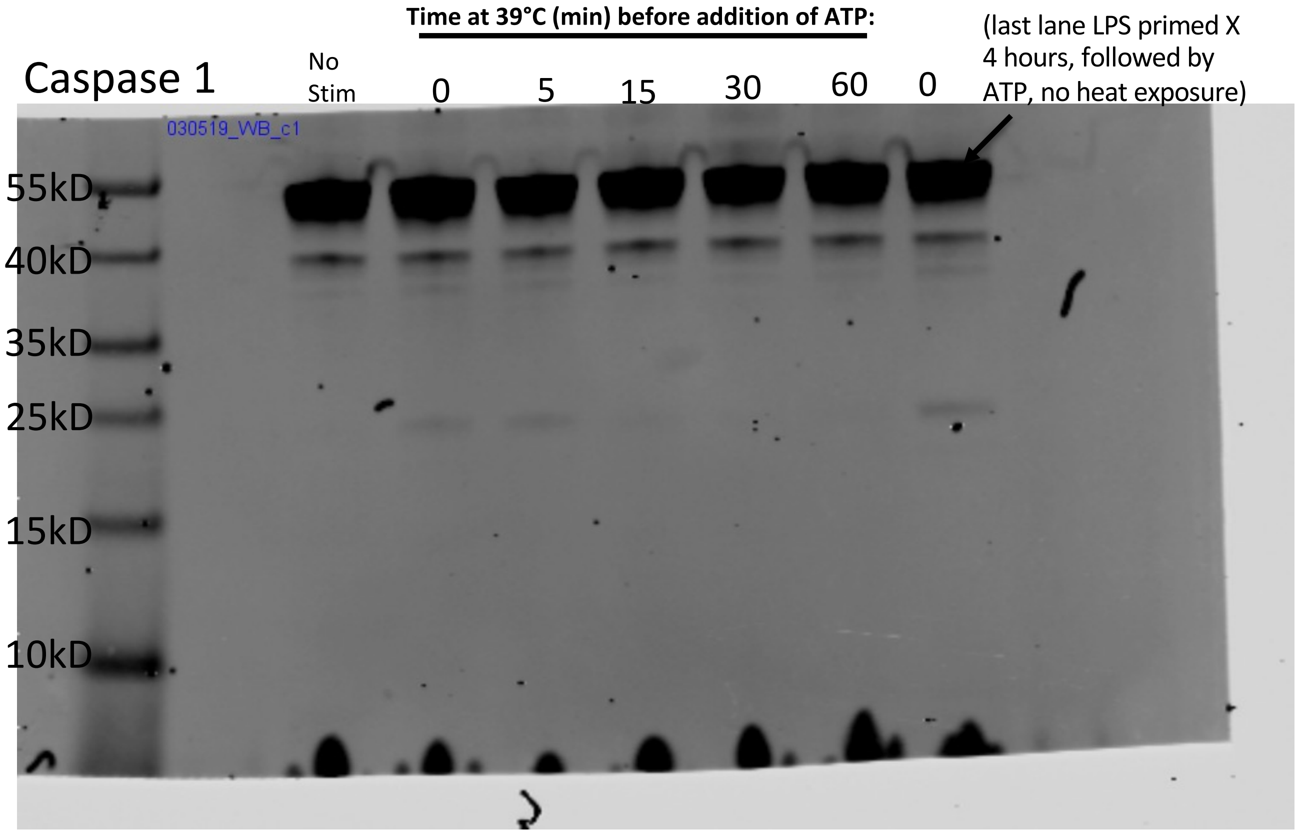

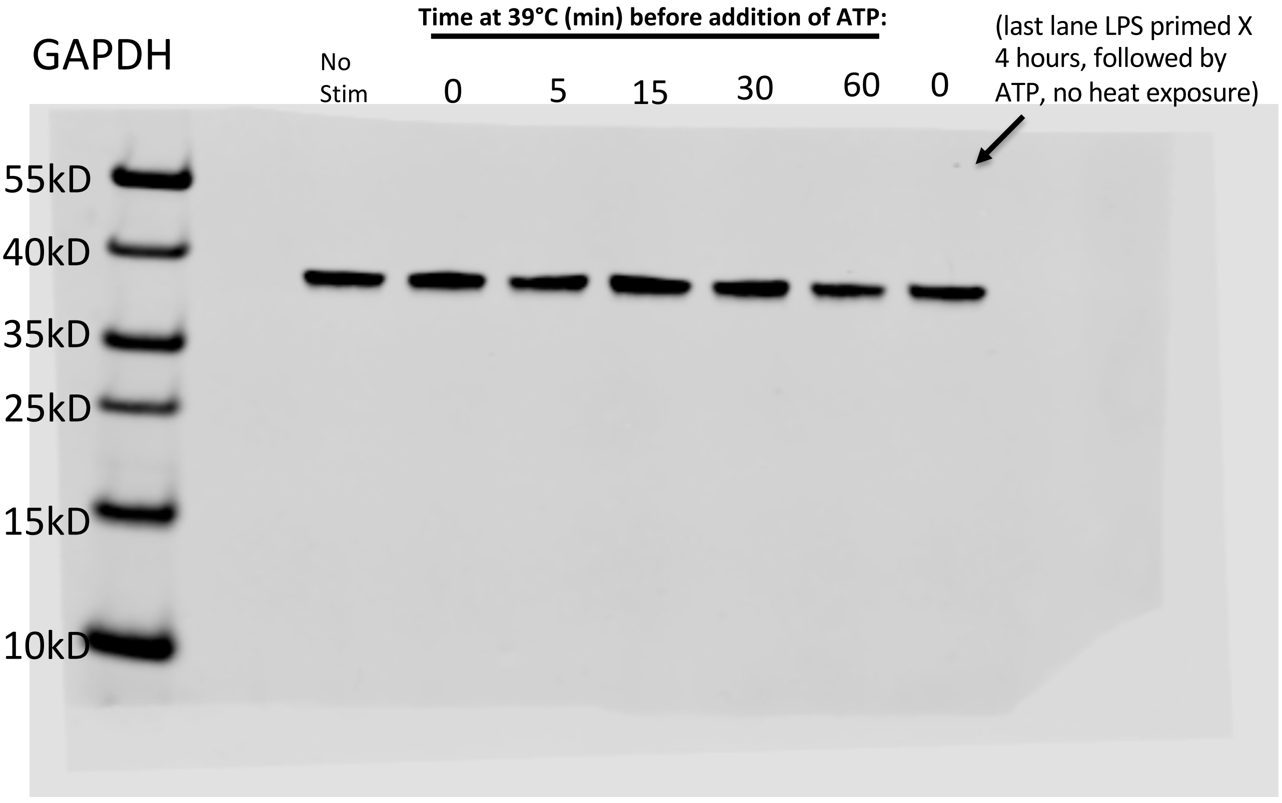
GAPDH
Time at 39°C (min) before addition of ATP:
No Stim
0
5
15
30
60
0
(last lane LPS primed X 4 hours, followed by ATP, no heat exposure)
55kD
40kD
35kD
25kD
15kD
10kD

Timecourse 2: IL-1β

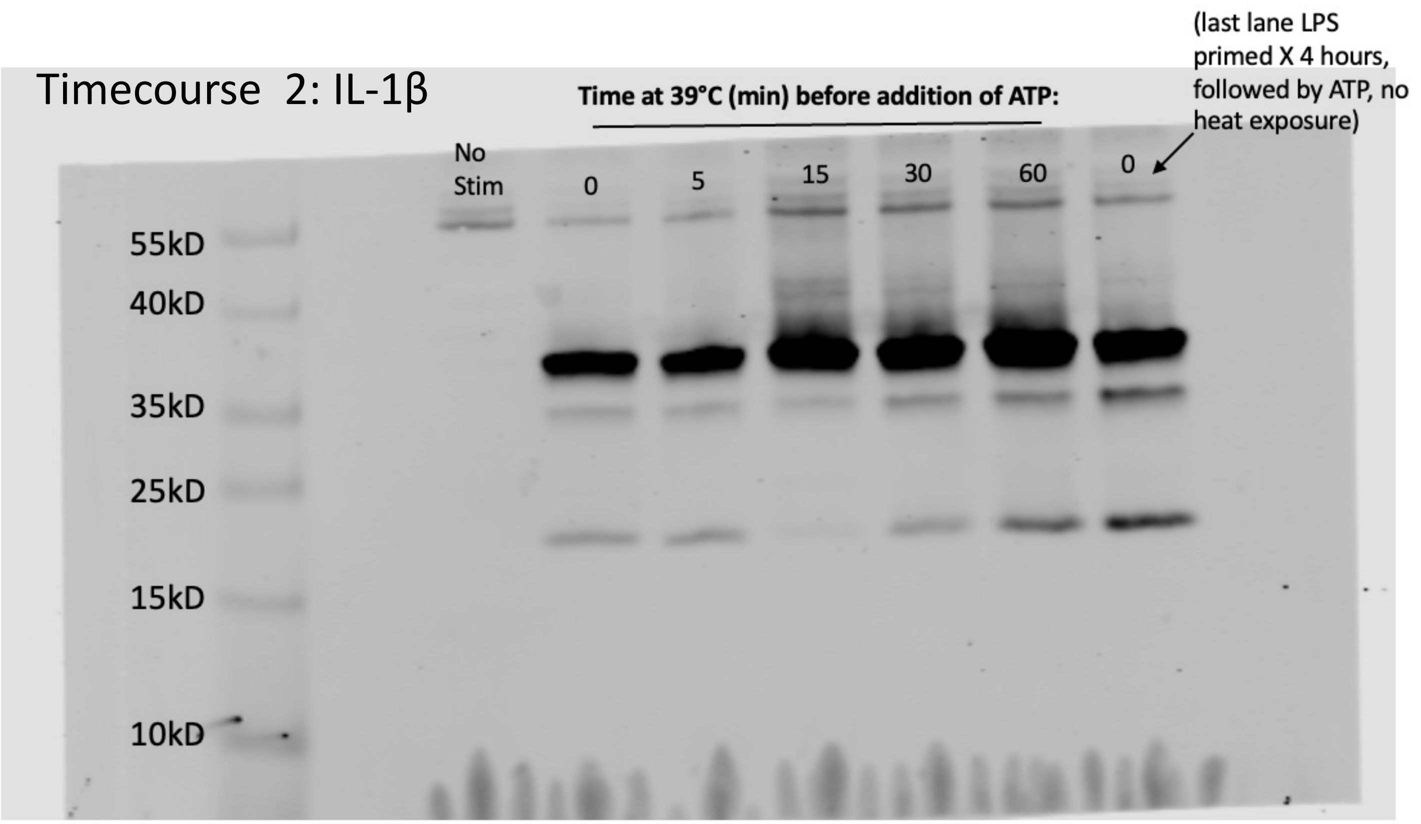

Timecourse 2: Caspase 1

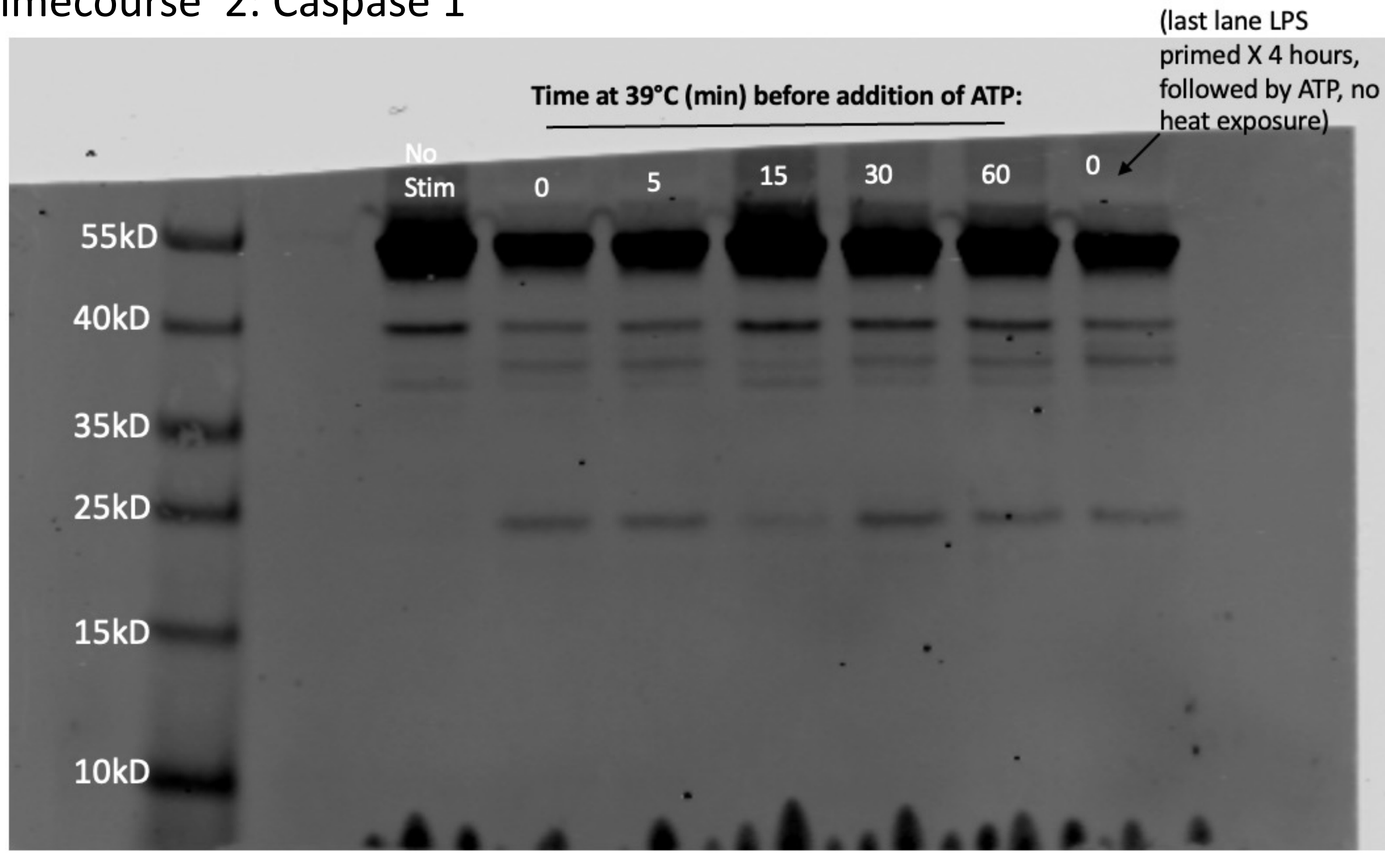

Timecourse 2: GAPDH

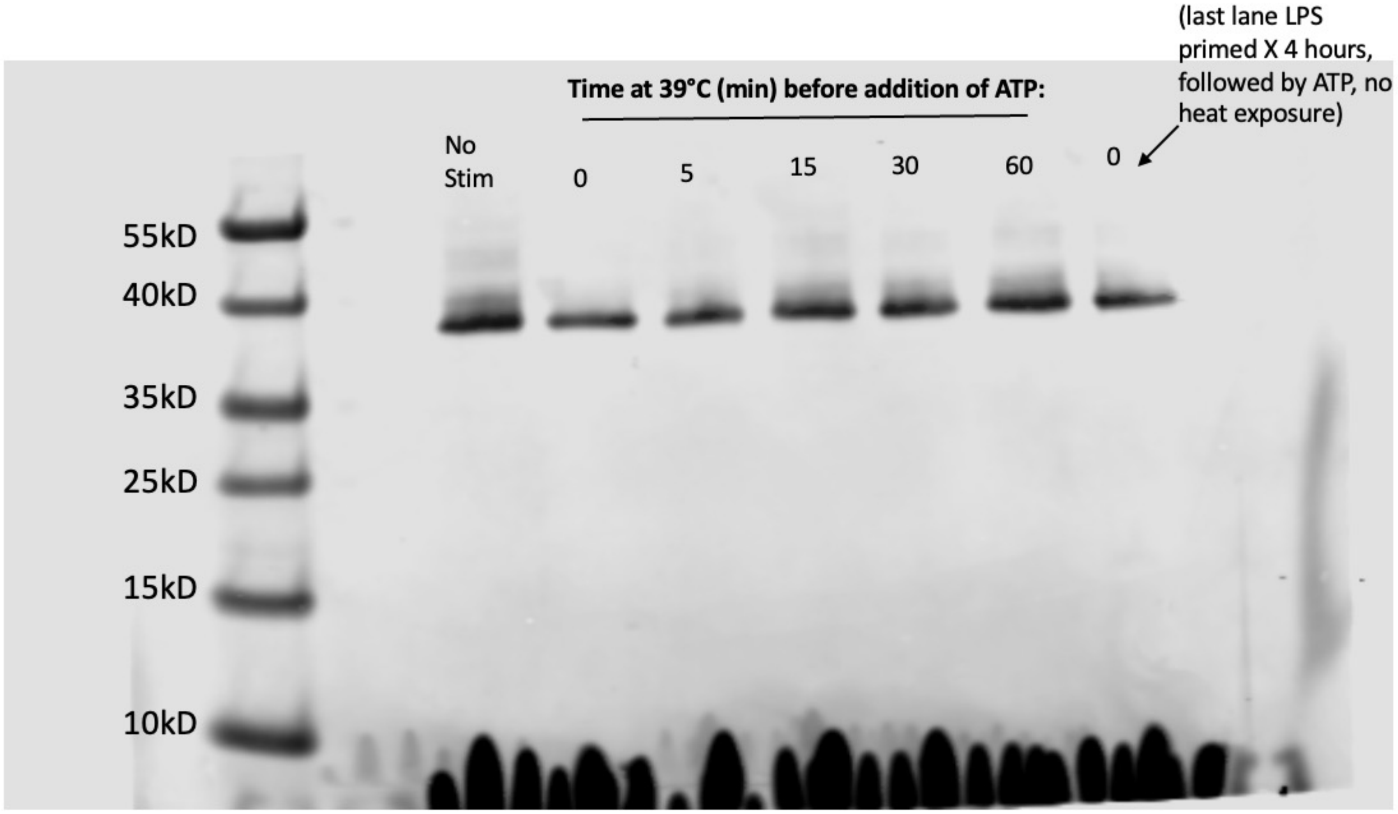

Supplemental FIGURE S4

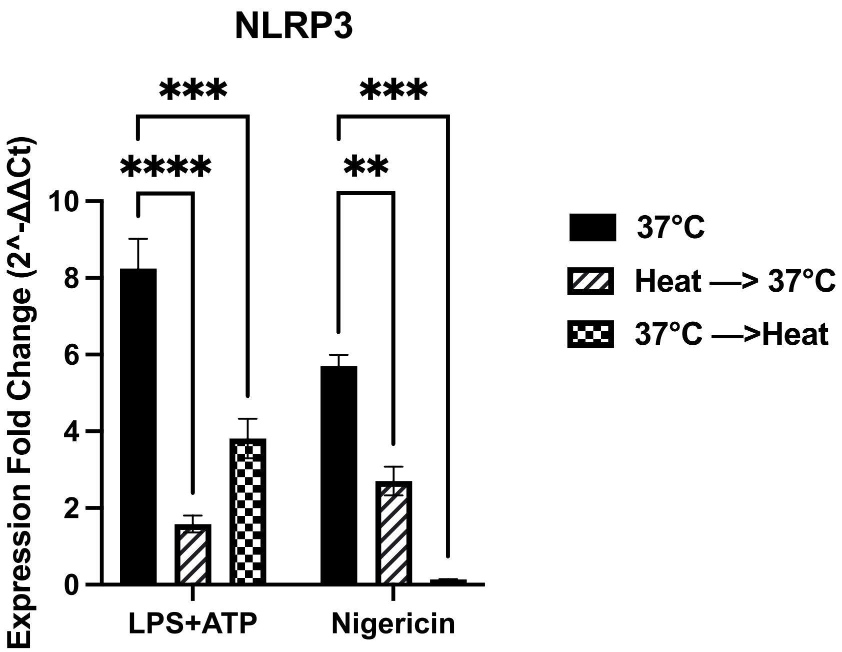

Figure S4. Exposure to heat shock inhibits transcription of NLRP3. Cells were treated with NLRP3 activating stimuli (LPS+ATP or Nigericin) and incubated at 37°C for 4 hours(black bars), exposed to heat shock for one hour followed by incubation at 37°C for 3 hours (hatched bars), or incubated at 37°C for 3 hours followed by heat shock for 1 hour (checked bars). Results are expressed as fold change over unstimulated and normalized to GAPDH.

Supplemental FIGURE S5

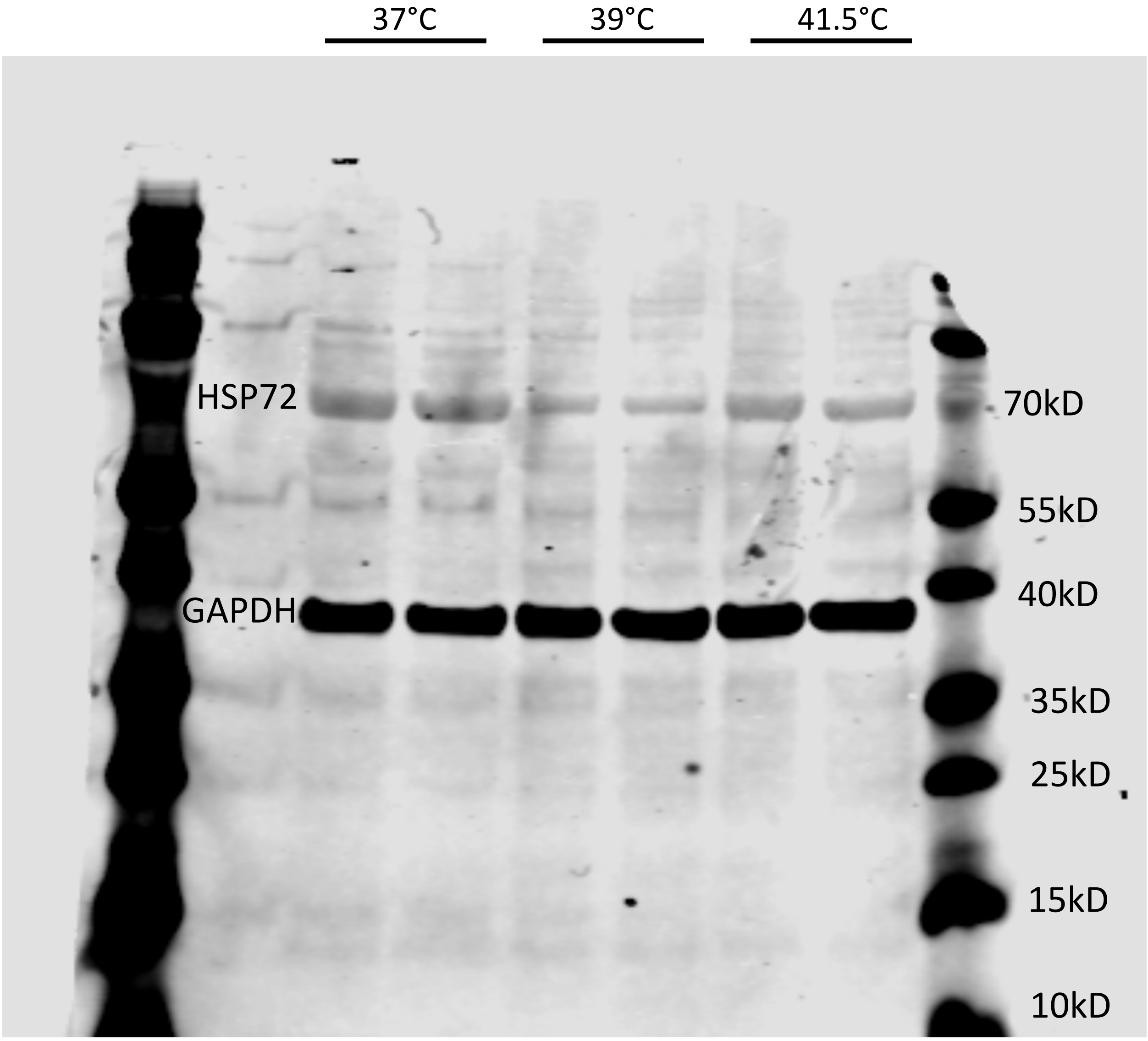

Figure S5. Western blot for HSP72 shows constitutive expression at 37°C and no induction after 1 hour of exposure to 39°C or 41.5°C. Results are representative of 2 independent experiments.
